# The Mechanical and Thermal Properties of Poly(ethylene-*co*-vinyl acetate) (PECoVA) Composites with Pristine Dolomite and Organophilic Microcrystalline Dolomite (OMCD)

**DOI:** 10.3390/polym13183034

**Published:** 2021-09-08

**Authors:** Lim Kean Chong, Azlin Fazlina Osman, Asfa Amalia Ahmad Fauzi, Awad A. Alrashdi, Khairul Anwar Abdul Halim

**Affiliations:** 1 Faculty of Chemical Engineering Technology, University Malaysia Perlis (UniMAP), Arau 02600, Perlis, Malaysia; keanchong97@gmail.com (L.K.C.); asfauzi18@gmail.com (A.A.A.F.); kanwar@unimap.edu.my (K.A.A.H.); 2 Biomedical and Nanotechnology Research Group, Center of Excellence Geopolymer and Green Technology (CEGeoGTech), Universiti Malaysia Perlis (UniMAP), Arau 02600, Perlis, Malaysia; 3 Chemistry Department, Umm Al-Qura University, Al-Qunfudah University College, Al-Qunfudah Center for Scientific Research (QCSR), Al Qunfudah 21962, Saudi Arabia; aarashdi@uqu.edu.sa

**Keywords:** dolomite, poly(ethylene-co-vinyl acetate) (PECoVA), composites, mechanical properties, thermal properties

## Abstract

Poly(ethylene-co-vinyl acetate) (PECoVA) composite containing organophilic microcrystalline dolomite (OMCD) was studied to replace the non-recyclable silicone elastomer in biomedical application. Pristine dolomite (DOL) is an inorganic mineral filler and is hydrophilic in nature, hence incompatible with most polymers and limits its use in biomedical applications. DOL was subjected to a combination of size reduction, tip sonication and a surface modification process to obtain a more effective dolomite filler, known as OMCD, as reinforcement material in the PECoVA copolymer matrix. The effects of DOL and OMCD loadings (1, 3, 5 wt%) on the structure and properties of the PECoVA composite were investigated. According to the X-ray diffraction (XRD), field emission scanning electron microscopy (FESEM), tensile and tear tests, dynamic mechanical analysis (DMA) and differential scanning calorimetry (DSC) analysis, the use of the OMCD filler brought a more pronounced positive impact to the PECoVA matrix as opposed to the DOL, where it enhanced the crystallinity of the matrix and led to much better matrix–filler interfacial interactions. Therefore, regardless of the filler loading, the PECoVA/OMCD composites demonstrate greater mechanical and thermal properties compared to the PECoVA/DOL composites. The best composite was produced with the OMCD loading of 3 wt%, in which the tensile strength (22.1 MPa), elongation at break (1413%) and Young’s modulus (2.0 MPa) of the copolymer matrix were increased by 44%, 23% and 21%, respectively. This proved that the combination of size reduction, tip sonication and the surface modification technique is efficient to obtain the PECoVA/dolomite composite with improved performance.

## 1. Introduction

For the last couple of decades, developments in the man-made devices or implants for biomedical procedures have led to the growth of the biomaterial-based healthcare industry. The unique and wide range of properties of polymer allow many researchers to explore and use it in biomedical applications. Nowadays, the most flexible and widely used biomaterials type is represented by polymers [1]. The polymers used in biomedical applications should be biocompatible and non-toxic because they have direct interaction with the human body and thus need to follow the essential requirements for which they are intended, such as comfort ability and ability to protect the human tissues [2]. In a few research papers, the development of poly(ethylene-*co*-vinyl acetate) (PECoVA) copolymer blends and composites for biomedical applications has been documented. A research conducted by Osman et al. [3] reported the use of PECoVA as insulation materials, due to their potential to replace non-recyclable silicone elastomer for implant biomedical devices applications.

PECoVA copolymers have recently gained great interest because of their properties, which can be obtained by varying vinyl acetate (VA) content. In this research, a rubber-like thermoplastic type of PECoVA with 21% VA composition was used as matrix material to form polymer composites. PECoVA is a random copolymer of ethylene and VA monomer. The higher the weight percent of VA, the higher the rubber-like properties of the PECoVA. The reasons for the recognition of PECoVA as a biomedical material are that it is not carcinogenic, has no adverse impact on human health and is biocompatible with human tissues [3,4,5].

Dolomite is a sedimentary carbonate rock which consists of calcium (Ca), magnesium (Mg), carbon (C) and oxygen (O_2_) elements. The structure of dolomite is in a crystal lattice form and consists of three layers, where the alternating layers of calcium and magnesium are separated by a carbonate (CO_3_) layer which gives a chemical formula of Ca·Mg(CO_3_)_2_ [6]. High quality dolomite has been discovered in Perlis, Malaysia in 1985 [7]. This dolomite has long been used as concrete aggregate and a source of magnesium and calcium, since this type of mineral stone is rich in these metal elements. However, our current study is the first attempt to use the Perlis’s dolomite as mineral filler in the PECoVA composite system, particularly for biomedical application.

In the production of the polymer/dolomite composite, the good mixing between the polymer matrix and dolomite filler will determine the properties of the produced composites [8,9]. Therefore, reduction of particle size of dolomite is crucial to increase its surface area and facilitate its dispersion and distribution inside the polymer and copolymer matrices. Furthermore, a chemical modification on the surface of dolomite should be considered since inorganic filler have poor contact with organic type polymer matrix [8]. The dolomite particles can be disaggregated and distributed into individual micrometer size particles when an ideal chemical modification is applied. The ideal chemical modification can promote a better interfacial interaction between the dolomite particles and copolymer matrix molecular chains. Dolomite particles with different organic modifications can be optimized to be compatible with various polymer systems [10].

The potential of dolomite to improve the properties of polymer matrices have been proven in various studies [9,11,12,13,14]. Ankabi et al. employed a grafting method to improve the mechanical properties of the polypropylene/dolomite composite. The research involved the use of polypropylene-grafted maleic anhydrate (PP-*g*-MA) with a small amount of compatibilizer [11]. Results showed that there was an improvement in mechanical properties when 20% of dolomite was used as filler. Tensile strength of the polypropylene was increased by 37%. This research revealed that the use of compatibilizer can enhance the interfacial adhesion between the polymer and dolomite filler. Another research study indicates the possibility to use of using dolomite as filler in enhancing the mechanical properties of the phenolic composite. The micro-hardness number of the phenolic composite can be increased with the addition of 28 wt% dolomite [12]. In a research where polyester and dolomite were employed as matrix and filler, respectively, the resultant composite possessed greater mechanical properties as opposed to the neat polyester. In addition, modulus and hardness values were successfully increased when the dolomite was added in higher loading [13]. Nik Adik et al. reported that thermal stability of polypropylene was enhanced when reinforced with 25 wt% dolomite filler that has been treated with stearic acid [14]. This proved that when the filler is chemically modified, well-blended filler and polymer matrix can be achieved and homogeneous polymer composite is formed. Thus, the polymer composite with the treated dolomite may exhibit greater thermal stability than one the composite with the untreated dolomite. Lastly, in the most recent publication, dolomite filler has been proven to significantly improve the tensile and tear strength of the thermoplastic starch film, showing that this mineral filler is also capable to enhance the mechanical properties of the natural or bio-based polymers [9].

In this study, the dolomite which underwent size reduction through ball milling and tip sonication process, followed by surface modification using stearic acid, was employed as filler in the production of PECoVA composite. It is referred to as organophilic-modified microcrystalline dolomite (OMCD). The PECoVA/dolomite composites with different OMCD loading were produced and studied based on their structure, thermal and mechanical properties. The control samples of PECoVA/dolomite composites with different loadings of pristine dolomite (DOL) were also prepared and tested in order to realize the effectiveness of the size reduction and surface modification combination method in enhancing the mechanical and thermal properties of the PECoVA composite.

## 2. Materials and Methods

### 2.1. Materials 

The PECoVA with 21% vinyl acetate (VA) composition was manufactured by Hanwha Total Petrochemical Co., Ltd. (Seosan-si, Korea). Dolomite was supplied by Perlis Dolomite Industries Sdn. Bhd. (Padang Besar, Malaysia). Stearic acid and Isopropyl alcohol (2-propanol) were manufactured by HmbG Chemicals (Hamburg, Germany) and purchased through A.R Alatan Sains Sdn. Bhd. (Alor Setar, Malaysia).

### 2.2. Preparation of Size Reduced Microcrystalline Dolomite

The combination of planetary ball milling and a tip sonication process was applied to obtain dolomite particles with a size reduced microcrystalline structure. First, the dolomite in powder form with 150 µm size was grinded into finer and small particle size by using the planetary ball mill with equipment model of PULVERISETTE 6 classic line (FRITSCH) (Bayern, Germany) for 10 h, with 19 repetitions at 500 rpm. The ball mill machine was initially filled with 50 stainless steel balls of 15 mm diameter. Then, the Digital Ultrasonic Disrupter model BRANSON 450 with lower section titanium micro tip (Queensland, Australia) was employed to provide ultrasonic shear effect for disaggregating and dispersing the dolomite particles using the tip sonication method. The dolomite:water ratio to form dolomite suspension was 1:10 (10 g of ball milled dolomite powder was added into 100 mL of distilled water). Dolomite suspension underwent the tip sonication process for 2 h with 30% of amplitude, while the pulse on value was 15 s and pulse off value was 10 s.

### 2.3. Surface Treatment of Microcrystalline Dolomite

The surface treatment of the size-reduced microcrystalline dolomite was performed according to the following procedures. The surface modifier aqueous solution was prepared by dissolving 2 wt% of stearic acid into 10 mL of isopropyl alcohol. The aqueous solution was stirred for 3 min at 50 °C, then transferred into the microcrystalline dolomite suspension and continuously mixed for 3 h using the IKA RW20 digital overhead stirrer machine with 3-bladed propeller stirrer (Alor Setar, Malaysia), under the speed of 400 rpm. Next, the surface-modified microcrystalline dolomite suspension was centrifuged for 10 min at 4000 rpm by using the Hettich Rotofix 32A benchtop centrifuge machine (Tuttlingen, Germany). After the sedimentation, the surface-modified microcrystalline dolomite suspension was poured into a petri dish and dried in an UF55 Plus universal oven (Memmert) (Büchenbach, Germany) for 24 h at 80 °C. The surface-modified microcrystalline dolomite powder was grinded by using mortar and pestle then sieved by using a 50 µm sieve. The resultant powder was ready to be used as organophilic microcrystalline dolomite (OMCD) filler.

### 2.4. Preparation of PECoVA/DOL and PECoVA/OMCD Composites

The PECoVA/OMCD composites were prepared with different OMCD loadings (1, 3 and 5 wt%). Control sample of the PECoVA composite with pristine dolomite (DOL) was also prepared. Table 1 shows the formulation of PECoVA/DOL and PECoVA/OMCD composites prepared for this study. Firstly, the PECoVA copolymer pellets and organophilic-modified microcrystalline dolomite (OMCD) powder was dried inside a Jeio Tech OV-12 vacuum oven (Rawang, Malaysia) under −0.06 MPa vacuumed condition, at a temperature of 50 °C for 24 h, to remove the water content prior to the melt compounding process. This is to ensure that melt mixing process between the PECoVA and OMCD in the heated two-roll mill can be optimized without the interference of moisture that can reduce the quality of the composites. The PECoVA pellets were mixed with OMCD by using the laboratory heated two-roll mill model DW5110, FYI (Hefei, China) at 160 °C, with front and back roller frequency of 4.65 and 3.0 Hz, respectively, and a mixing time of 20 min. The ratio of the peripheral speeds of the rolls, known as friction ratio, ranges from 1 to 2. The total gap between the rollers was set at 0.01 mm. When the heating temperature reached the set temperature, the pre-weighed PECoVA copolymer pellets were charged to the nip between two rollers for 5 min preheat. After preheating, both rollers started to roll the PECoVA melt and the OMCD powder was added into a rolling “bank” formed between the rollers. During the operation, cutting of the sheet of the composites’ melt “blanket”, folding and rolling were carried out by using a scraper, which increased the uniformity of the composition. The compounded PECoVA/OMCD composite was collected at the end of the process and cut into strips. The same procedures were performed for the sample preparation of the PECoVA composite with DOL.

### 2.5. Compression Moulding

The composite sheet samples with one millimeter thickness were prepared by using the electrically-heated hydraulic-press compression molding machine model GT 7014 A, GOTECH (Taichung City, Taiwan). The standard practice for compression molding involved pre heating the mold with dimensions of 150 mm× 150 mm× 1 mm (length × width × thickness) at 160 °C for 3 min, followed by compressing the 22.5 g of composite strips for 5 min with 10 kPa pressure at the same temperature. The pre-heating step was done to ensure uniform heat flow throughout the material. The samples were cooled under pressure for another 7 min before being withdrawn from the mold. 

### 2.6. Scanning Electron Microscope (SEM) Analysis

The morphology and particle size of the dolomite (before and after being ball milled and tip sonicated) were examined through SEM with the equipment model number of JEOL JSM-6460LA (JEOL. Ltd., Tokyo, Japan). In addition, the success of surface modification was also confirmed by analyzing the surface morphology of the OMCD filler compared with the DOL filler. The powder sample was prepared by collecting a small amount of filler powder with a spoon and letting it fall on a carbon double-sided sticker, then using spray air to remove the excess particles. A thin layer of platinum was applied to coat the sample in order to enhance the quality of the captured images. The powder sample together with the sticker were mounted rigidly on an aluminum pin stubs, then sputter-coated with a thin layer of palladium. This was to avoid electrostatic charging during characterization. The magnifications used were at 500× and 1000×.

### 2.7. Field Emission Scanning Electron Microscopy (FESEM) Analysis

The tensile-fractured surface morphology of the virgin PECoVA, PECoVA/DOL composite and PECoVA/OMCD composite was examined through field emission scanning electron microscopy (FESEM), with the equipment model number of ZEISS LEO 1525 (ZEISS, Oberkochen, Germany) by referring ASTM F1877-98(2003). A thin layer of platinum was employed as the coating layer to allow the capture of good images. The tensile fracture end of the sample was mounted rigidly on aluminum pin stubs then sputter-coated with a thin layer of palladium. This was to avoid electrostatic charging during characterization. The magnifications used were 500×, 1000× and 2500×.

### 2.8. Fourier-Transform Infrared Spectroscopy (FTIR) Analysis

The FTIR analysis of the pristine dolomite (DOL) and organophilic microcrystalline dolomite (OMCD) filler was performed by using the Perkin Elmer RXI FTIR spectrophotometer (Waltham, MA, USA). The sample was analyzed by using the potassium bromide (KBr) pellet method. The FTIR spectra for the OMCD filler were compared with the unmodified DOL filler for the purpose of analyzing the presence of new chemical functional groups. The samples were recorded for 32 scans in the frequency range of 4000–650 cm^−1^ wavenumber with resolution of 32 cm^−1^.

### 2.9. X-ray Diffraction (XRD)

The crystallinity of DOL filler, OMCD filler, virgin PECoVA, PECoVA/DOL composite and PECoVA/OMCD composite was characterized by using X-ray diffraction (XRD) with the model Bruker D2 Phaser (Billerica, MA, USA), with Cu Kα radiation and the wavelength, λ, of 1.5406 Å. The voltage used was 35 kV with the current of 25 mA. The scattering angle range 2θ used was 10°–90°, while the step size and the time per step were 0.022 and 19.2 s, respectively. The crystallinity index or peak-to-noise ratio of the samples, *CI_XRD_* (%), was calculated using Equation (1):

(1) $$CI_{XRD}\;\left(\%\right)=\frac{I_{C}}{\left(I_{C}+I_{A}\right)}\times 100\%$$

where *I_C_* represents the area of crystalline peaks of the sample, which is obtained by calculating the area under the crystalline peaks and *(I_C_* + *I_A_)* is the total area under all of the peaks of the sample.

### 2.10. Tensile Test

A dumbbell cutter machine was used to cut the composite sheets into dumbbell-shape specimens according to the dimension specified in ASTM D638 Type V. Tensile test of the virgin PECoVA, PECoVA/DOL and PECoVA/OMCD composites was conducted by using the Universal Testing Machine Instron 5569 (Norwood, MA, USA) to determine their tensile properties according to ASTM D638. Tensile load was applied using cross-head speed of 50 mm/min. The results of five replicates of each sample were recorded at the end of each test and the average values of tensile strength, elongation at break (Eb) and Young’s modulus were obtained for comparison between samples.

### 2.11. Tear Test

The tear strength of the virgin PECoVA, PECoVA/DOL and PECoVA/OMCD composites was determined by using Universal Testing Machine Instron Machine 5569 (Norwood, MA, USA) according to ASTM D624. The samples were cut according to angle test specimen (ASTM D624 Type C) and the test was run at crosshead of 500 mm/min. The results of five replicates of each sample were recorded at the end of each test and average values of tear strength were taken.

### 2.12. Differential Scanning Calorimetry (DSC) Analysis

Differential scanning calorimetry (DSC) model DSCQ-10 by TA instruments (New Castle, Delaware, United States) was used to measure thermal behaviors of the virgin PECoVA, PECoVA/DOL and PECoVA/OMCD composites. The weight of the sample was about 5 mg. Next, the heating rate was set at 10 °C/min and the atmosphere was purged with nitrogen gas, with the gas flow rate of 50 mL/min, in order to get the melting temperature (T_m_) and the melting enthalpy (Δ*H*) of the samples. The samples were then heated from room temperature (30 °C) to 150 °C. The degree of crystallinity of the samples, *X_C_*, was calculated using Equation (2) below:

(2) $$X_{C}\;\left(\%\right)=\frac{\Delta H_{f}}{\Delta H_{0}}\times 100\%$$

where Δ*H_f_* represents the melting enthalpy of the sample, which is obtained by calculating the second heating endothermic peak, and Δ*H*_0_ is the theoretical melting enthalpy of the 100% crystalline polymer. In this case, the Δ*H*_0_ of PECoVA is approximately 23.47 J/g.

### 2.13. Dynamic Mechanical Analysis (DMA)

The Perkin Elmer Pyris Diamond DMA (Waltham, MA, USA) was employed to determine the viscoelastic properties of the virgin PECoVA, PECoVA/DOL and PECoVA/OMCD composites. The obtained data were used to characterize the elastic reaction deformation and analyze the damping behavior, which is useful for determining the occurrence of molecular mobility transition. Analysis was performed at 0.1% strain in bending mode using a frequency of 1 Hz and a heating rate of 2 °C/min from room temperature to 90 °C.

## 3. Results and Discussion

### 3.1. Morphological Analysis of Pristine Dolomite (DOL) and Organophilic Microcrystalline Dolomite (OMCD) by Scanning Electron Microscopy (SEM)

**Figure 1:**
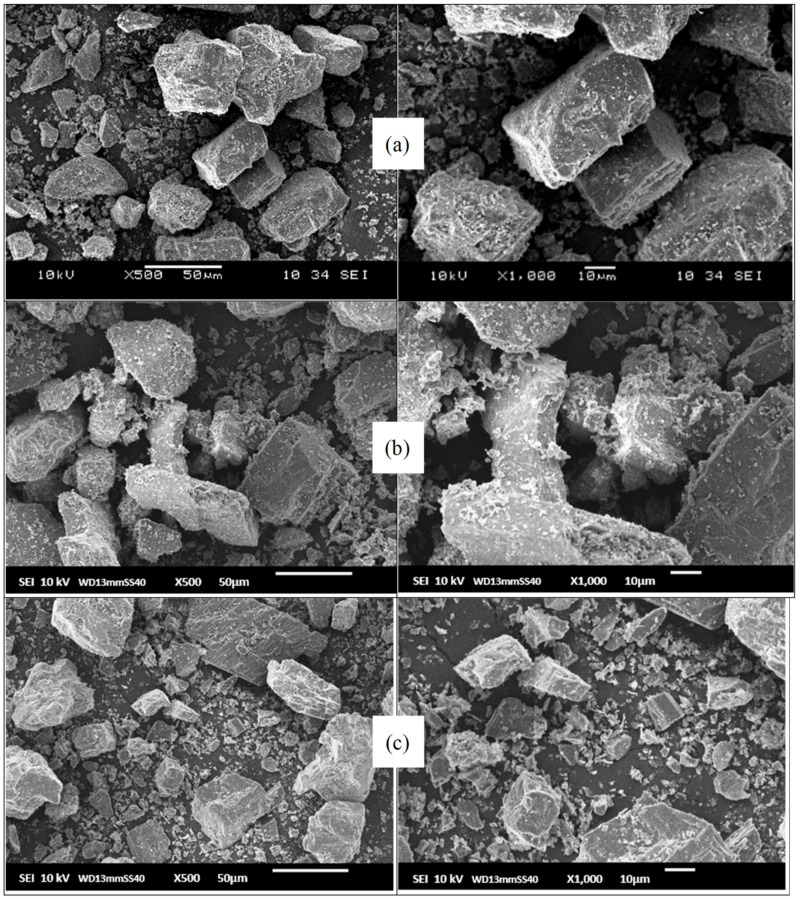
SEM images of filler particles of (**a**) DOL, (**b**) DOL+10H and (**c**) DOL+10H+2H at 500× magnification (**left**) and 1000× magnification (**right**).

SEM analysis was performed to observe the morphology and compare the particle size of the dolomite (before and after being ball milled and tip sonicated). Figure 1 presents the SEM micrographs of the filler particles of the pristine dolomite (DOL), DOL with 10 h planetary ball milling (DOL+10H) and DOL with 10 h planetary ball milling and 2 h tip sonication (DOL+10H+2H) at 500× magnification (left) and 1000× magnification (right). Table 2 shows the surface area and particle size of all the materials. In the pristine form of dolomite (DOL), the average particles size was ~58 µm. However, upon 10 h of planetary ball milling process, the particles of the DOL became smaller and finer. The average particle size of the DOL filler was decreased from 58 to 29 μm after the planetary ball milling process of 10 h, as shown in Figure 1a,b, respectively. This implies that the ball milling process can be used to reduce the particles size of DOL. However, the SEM diagram suggests that the microcrystalline particles of DOL tends to aggregate together into large size particles. Agglomeration of filler usually occurs after being dried from the suspension form [9].

By comparing Figure 1b,c, we can prove that the particle size and agglomeration of the (DOL+10H) have been reduced significantly upon the tip sonication process. Before tip sonication (Figure 1b), the (DOL+10H) contains large particles with an average particle size of 29 μm. This was due to the high crystal–crystal adhesion of DOL, leading to the crystalline agglomerates structure. After being tip sonicated (Figure 1c), the particle size of the DOL was seen to greatly reduce to 15 μm, with rougher crystal surface morphology and edges. It was postulated that the tip sonication procedure removed most of the small particles attached and grown on the surface of large crystals, and at the same time broke down the agglomerates of equally sized particles. This could be achieved because the ultrasound energy has the ability to break some of the agglomerates, leading to the fracturing of the dolomite crystals [15].

Previous works by Sander, Zeiger and Suslick were aimed to investigate the effect of sonication on crystal slurries in order to identify the mechanism of crystal fragmentation by ultrasound [16,17]. They have concluded that the interaction between particles and cavitation shockwaves is the primary cause for this phenomenon, while other potential sources, such as inter-particle, particle–horn and particle–wall collisions, only marginally contribute to the fragmentation process. Wagterveld et al. have successfully visualized these events by the use of high-speed imaging during sonication of calcite crystals [18]. At the end of the experiment, the ultrasonic treatment yielded smaller particles with rough crystal surfaces and edges, besides a significant amount of fines. The results confirmed that the tip sonication process has successfully reduced the agglomeration and crystalline structure of the dolomite. These findings support our SEM and mechanical test results whereby the addition of the OMCD filler in the PECoVA matrix resulted in a more reinforced composite due to the size reduction of the microcrystalline filler. It is well understood that the smaller size filler can be more easily penetrated and distributed in the free volume of the polymer matrix [9].

### 3.2. Chemistry Analysis of the DOL and OMCD by Fourier-Transform Infrared Spectroscopy (FTIR)

**Figure 2:**
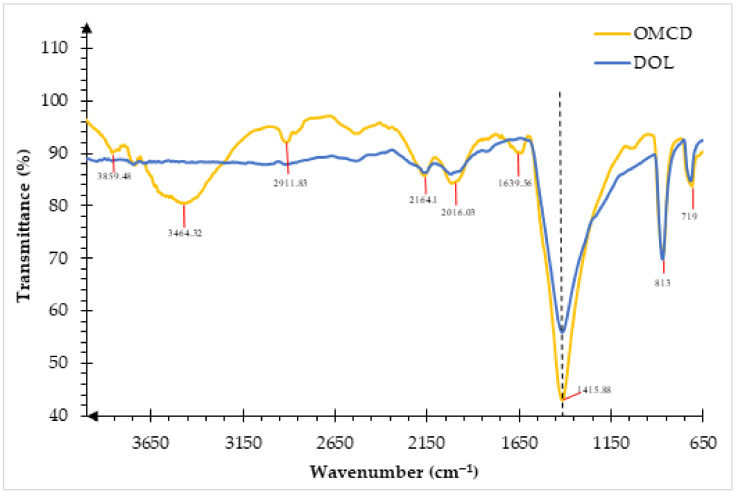
FTIR spectra of DOL and OMCD filler.

Fourier-transform infrared spectroscopy (FTIR) analysis was conducted on the DOL and OMCD fillers. The FTIR spectra of the DOL and OMCD are shown in Figure 2. Infrared (IR) absorption takes place when the emission spectra frequency matches the vibrational frequency of the bond. Light frequency can be detected in two ways: transmission or reflection. The detection mode used this studied was transmission where the amount of light frequency that passes through the sample was detected and showed in the FTIR graph.

In general, the FTIR spectra of DOL and OMCD reveal that carbonate is the most major constituent in dolomite [19]. The magnesium carbonate (Mg_2_CO_3_) and calcium carbonate (Ca_2_CO_3_) (carbonate mineral group) exhibited the strong peak at 1415.88 cm^−1^, which is associated with lattice CO_3_^2−^ [20]. The appearance of spectral peaks at 3000 and ~719 cm^−1^ revealed the other main components of dolomite [21]. The FTIR absorption at 3000 cm^−1^ relates to organic residue, while the band at 2870 cm^−1^ represents the calcite combination band. The peak at 719 cm^−1^ is attributed to magnesium calcite or calcite [9]. Ji et al. stated that the FTIR absorptions at 1420, 873, and 719 cm^−1^ proved the presence of dolomite, which also agrees with our results [22]. 

Then, the FTIR spectra of dolomite before (DOL) and after chemical treatment with stearic acid (OMCD) in the wavenumber range of 650 to 4000 cm^−1^ were compared. The main difference of the IR signal can be observed at the spectral peaks of 1639.56, 2911.83 and 3464.32 cm^−1^, which are assigned to the C–H stretching and O-H stretching vibration mode, as per discussed by Fuji et al. [23]. However, the peaks are not observed in the DOL spectra, because they are associated with the C–H and O–H stretching modes in the organic surfactant. The molecular formula of stearic acid, CH_3_(CH_2_)_16_COOH indicates that there is strong C–H and O–H bonding in the surface modifier, thus we can confirm that the surface modification process was successful. In addition, there are significant changes in the spectra peaks at 1639.56, 2911.83 and 3464.32 cm^−1^ when the dolomite is subjected to the physical and chemical modification processes. Thus, it is proven that the mineral filler has been significantly modified from its pristine state.

It is also worth noting that there are more intense peaks around 1415.88, 2016.03, 2164.18, and 712.27 cm^−1^ for the OMCD when compared with the DOL. This might be because the planetary ball mill and the tip sonication process reduced the particle size and increased the total surface area of the dolomite. It is widely known that the infrared (IR) spectroscopy involves the study on how the infrared light interacts with matter. Therefore, the concentration of molecules in the tested sample affects the peak intensity in the IR spectra [24]. The higher surface area of the filler particles allowed the IR light to penetrate into a greater number of molecules, resulting in the intense peaks observed. The main objective of using a planetary ball mill and tip sonication in this study was to break down and de-agglomerate the dolomite particles, into smaller and finer sizes, so that the dolomite filler could be dispersed more homogenously throughout the PECoVA matrix. Due to a better interaction between the dolomite filler and the PECoVA matrix, the tensile and tear properties could be improved.

### 3.3. Morphological Analysis of Virgin PECoVA, PECoVA/DOL Composite and PECoVA/OMCD Composite by Field Emission Scanning Electron Microscopy (FESEM)

**Figure 3:**
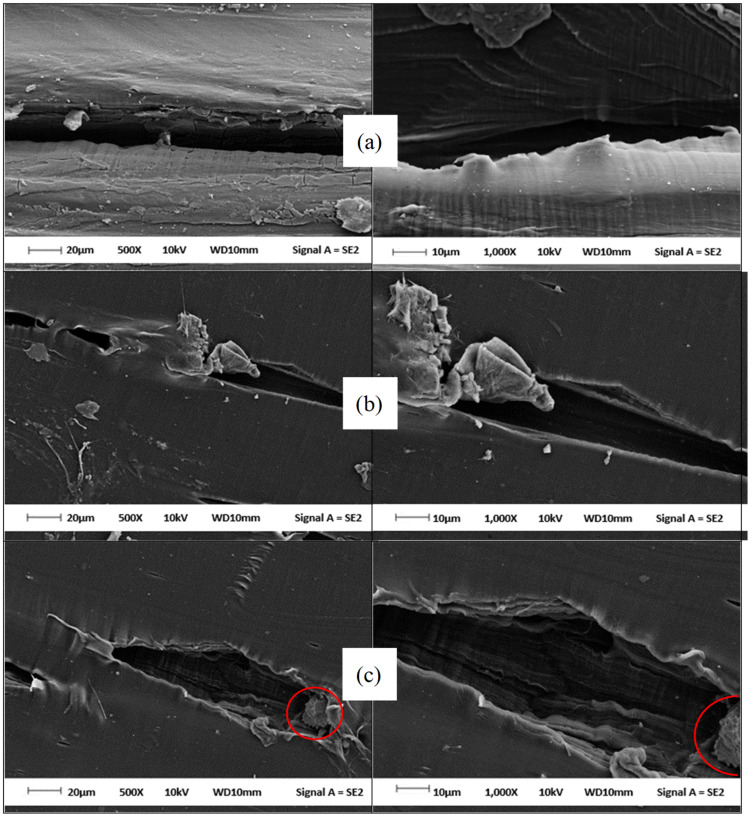
FESEM images of tensile-fractured surface of the (**a**) virgin PECoVA, (**b**) PECoVA/DOL3 and (**c**) PECoVA/OMCD3 composite at 500× magnification (**left**) and 1000× magnification (**right**).

The tensile-fractured surface morphology of the virgin PECoVA, PECoVA/DOL3 composites and PECoVA/OMCD3 composites were studied by field emission scanning electron microscopy (FESEM). The magnifications of 500× and 1000× were used to analyze the effect of size reduction and surface treatment on the morphology of PECoVA composites. FESEM micrographs of the virgin PECoVA, PECoVA containing pristine dolomite (DOL) and organophilic microcrystalline dolomite (OMCD) are shown in Figure 3a–c, respectively. Figure 3c indicates that the OMCD filler was embedded in the PECoVA matrix without any sign of aggregation. It seems that the PECoVA matrix covered the surface of the OMCD filler. These observations further confirmed the XRD results, where diffraction peaks of the OMCD can be detected in the composite sheet.

The tensile-fractured surface of the PECoVA composite with the DOL (Figure 3b) exhibits more significant and uneven matrix tearing. In comparison with the PECoVA/OMCD composite, the PECoVA/DOL composite showed more significant tearing with large polymer matrix deformation when tension force was applied. This indicates that the stress exerted on the PECoVA/DOL composite distributed unevenly due to the poor interfacial adhesion between the DOL filler and the PECoVA matrix. When a tension force was exerted on the PECoVA/DOL composite, the crack propagates along the boundary of the PECoVA and DOL filler interfaces. However, there is a gap between the PECoVA and DOL filler due to poor interfacial adhesion between both constituents. Therefore, the stress exerted on the PECoVA/DOL composite only passed by the DOL filler due to the absence of interaction between filler and polymer matrix. As illustrated in Figure 3b, the crystalline structure of DOL is dislocated and loosens out upon the fracture. Recently, it has been suggested that the presence of such a phenomenon is an indication of low filler–matrix interfacial adhesion [25,26]. Furthermore, the origin of agglomeration and in turn of poor dolomite dispersion state are caused by the hydrophilic–hydrophilic interactions. In contrary, when the dolomite has already been hydrophobized by chemical treatment (OMCD), the hydrophobic–hydrophobic interactions are responsible for chemical affinity and interfacial adhesion. Li et al. showed that the composite with a poor dispersion of filler indicated a rough fractured surface with the appearance of voids, suggesting the poor wetting of filler by the polymer matrix [27].

The tensile-fractured surface of the PECoVA/OMCD composites (Figure 3c) shows a more homogeneous and smoother surface after being deformed and fractured by tensile forces. This observation indicates that the PECoVA filled with OMCD allows for more stress being distributed between the matrix and filler. This could be due to the homogenous dispersion of OMCD in the matrix as well as the improved interfacial adhesion, leading to a uniform stress distribution. Li et al. found out that the homogeneous dispersion of filler can improve the mechanical and thermal properties of the host polymer [27]. These findings support our DMA and DSC results for the PECoVA/OMCD composites, which will be discussed in the next session. The improved interfacial adhesion might be due to the physical and chemical modification performed that increased the surface area and surface compatibility between the non-polar group of the PECoVA and non-polar (organophilic) surface of the OMCD filler. When a tensile force was exerted on the PECoVA/OMCD composite, a fibrous-like structure was observed inside the deformed cavity, which was suspected to be the PECoVA-wetted OMCD. The fibrous-like structure indicates that the OMCD filler has a strong interfacial interaction with the PECoVA matrix and adhered strongly to the PE chains before it was pulled out from the PECoVA matrix. The red circle in Figure 3c shows that the OMCD filler particles are still partly embedded and strongly adhered to the PECoVA matrix after the fracture. Furthermore, the PECoVA/OMCD composite exhibits less matrix deformation upon breaking under tension. Therefore, the tear lines for the PECoVA/OMCD composite look shorter and more homogeneous compared with the PECoVA/DOL composite. The shorter tear line observed in the PECoVA/OMCD composite indicates a smaller tearing effect occuring after incorporation of the OMCD filler. This is because the stress exerted on the PECoVA/OMCD composite can be distributed more homogeneously because of better mixing between the PECoVA matrix and OMCD filler. These support the reason why the tensile strength of the PECoVA/OMCD composite is higher than the PECoVA/DOL composite.

### 3.4. Crystallinity of the Fillers and Composites by X-ray Diffraction (XRD) Analysis

#### 3.4.1. Crystallinity Analysis of the Dolomite Filler (DOL and OMCD)

**Figure 4:**
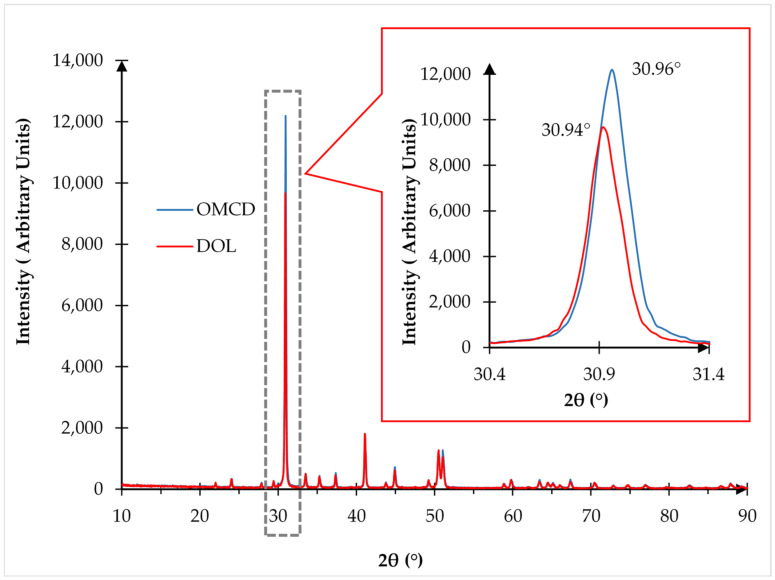
X-ray diffraction (XRD) pattern of PECoVA composites with different types of filler.

The structure of dolomite is ordered, thus its crystalline structure can be analyzed through XRD. The structures of the DOL and OMCD were characterized and compared by using XRD analysis. Figure 4 illustrates the XRD patterns of both types of filler.

The XRD pattern of dolomite (DOL and OMCD) reveals diffraction peaks at around 2θ = 30.9122° and 30.9517°, which are similar to that observed by other researchers [19,28,29]. However, when benchmarked with the DOL, the OMCD shows more intense diffraction peaks at both angles. This signifies that the crystallinity of dolomite was enhanced by the planetary ball milling and the tip sonication process. This can be explained by the reduction in particle size of OMCD filler has increased its surface area and allow itself to arrange more closely packed compare to DOL filler. As a result, signal from X-ray diffracted from the crystalline lattices of OMCD filler particles become stronger. To prove this, the degree of crystallinity of both types of dolomite was measured and tabulated in Table 3. The degree of crystallinity for DOL and OMCD was found to be 76.57% and 77.10%, respectively. The higher degree of crystallinity of the OMCD could be as a result of the 10 h planetary ball milling and 2 h sonication process. The reduction in particle size of dolomite has increased its surface area. As a result, signals from XRD that went through the crystalline lattices of dolomite particles became stronger [9].

#### 3.4.2. Crystallinity Analysis of the Virgin PECoVA, PECoVA/DOL Composite, and PECoVA/OMCD Composite

**Figure 5:**
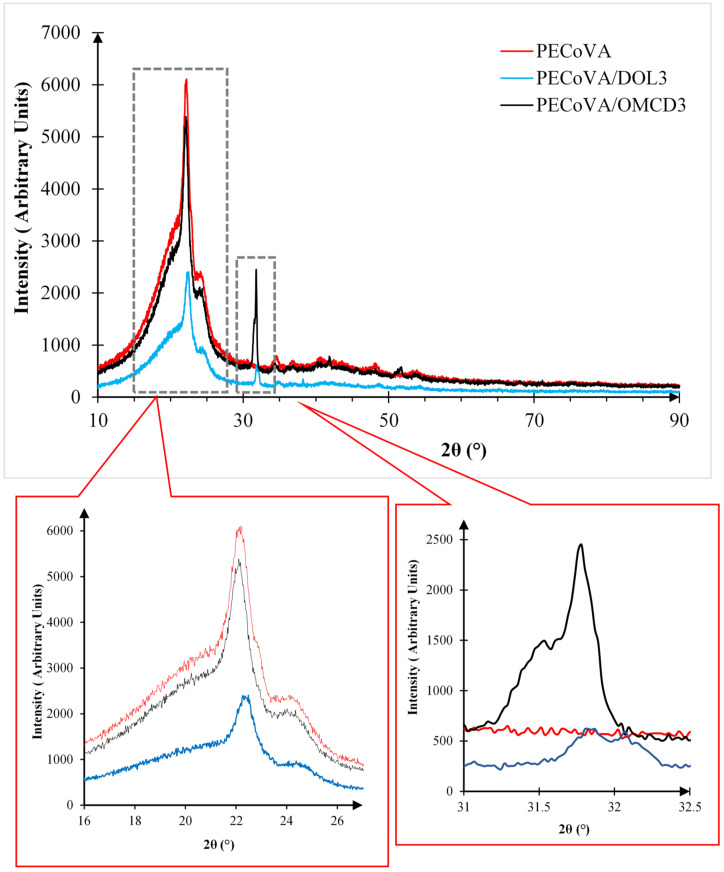
X-ray diffraction (XRD) pattern of PECoVA composites with different type of filler.

Figure 5 shows the results of XRD pattern of the virgin PECoVA, PECoVA/DOL3, and PECoVA/OMCD3 composite in the range of 10° to 90°. Analysis of the virgin PECoVA, PECoVA/ DOL3, and PECoVA/OMCD3 composite by XRD allowed the indexation of their diffraction spectra, by matching them with the PECoVA polymer spectrum of the equipment data library, as shown in Figure 5.

The most significant difference between the spectra corresponds to the position of the highest intensity peak, where for virgin PECoVA it is in the 2θ = 22.1930°, PECoVA/DOL in 2θ = 22.1930° and for PECoVA/OMCD in 2θ = 22.1040°. As the comparison, there was a match for dolomite (CaMg(CaCO3)2) in both composites samples. The peaks for this CaMg(CaCO3)2 substance are close to both of the PECoVA composites reflections at 31.7773°, 31.8408°, 41.8854°, 48.6021° and 51.7387°. Those dolomite correlated peaks proved the existence of dolomite particles in the structure of the PECoVA composite. Apparently, the peaks that exist in the both dolomite filler (DOL and OMCD) disappeared in the XRD signal of the PECoVA composites, which could be due to poor and uneven distribution of DOL in the copolymer matrix, leading to reduction in the diffraction signature. On the other hand, de-agglomeration of dolomite upon the tip sonication procedure enhanced the surface area of the dolomite particles, improving their dispersion in the matrix and thus allowing the detection of more intense diffraction signature.

PECoVA copolymer exhibits one sharp diffraction peak centered at 2θ = 22° and a broad shoulder at 2θ = 24° due to the crystalline structure of polyethylene (PE) [30]. The addition of DOL caused significant reduction in the intensity of both peaks, revealing that this filler, when it exists in the agglomerated form, may induce a greater thermodynamic barrier for the nucleation process of the ethylene phase. Large particles of DOL hinder the molecular motion of the ethylene phase, restricting the arrangement of the molecules into ordered structure. Upon the addition of dolomite into the PECoVA matrix, there was a crystalline peak detected at about 31.7° for both the PECoVA/DOL3 and PECoVA/OMCD3 composites. However, the most significant difference between the diffraction of the PECoVA/DOL3 and PECoVA/OMCD3 composites was the intensity of the diffraction being stronger in PECoVA/OMCD3 composites, which is affected by the collective diffraction of all the atoms in the crystal due to better dispersed of filler particles [9,31]. To support the statement, the degree of crystallinity of both types of composites was measured and is tabulated in Table 4.

The degrees of crystallinity for the virgin PECoVA, PECoVA/DOL3 and PECoVA/OMCD3 composites were found to be 23.29%, 23.69% and 34.72%, respectively. As referred to in Table 4, it can be seen that the addition of dolomite filler significantly increased the degree of crystallinity of the PECoVA. The degree of crystallinity of the virgin PECoVA was increased from 23.29% to 34.72% when incorporated with the OMCD. Dissimilarity between the crystallinity of the PECoVA/DOL3 and PECoVA/OMCD3 composites might be due to the particle size and homogeneity of the filler embedded in the free volume of PECoVA chains [31]. Apparently, the higher total surface area and more uniform dispersion of filler was observed in the PECoVA/OMCD3 composite, thus the highest degree of crystallinity achieved by this sample could be related to these factors [9]. De-agglomeration of dolomite upon the modification process leads to the increase of surface area and interfacial adhesion of the dolomite particles with the matrix phase. As a result, signals from X-ray diffraction that went through the cubic crystalline structure of the composite became stronger [4]. The increase of degree of crystallinity is commonly associated with the tensile strength improvement of the polymer matrices [9,31]. This is another reason why the PECoVA/OMCD3 composite has better tensile and tear strength when compared with the PECoVA/DOL3 composite, which will be shown and discussed in the next section.

The XRD analysis helped to identify the calcium carbonate (CaCO_3_) as a component of the sample. CaCO_3_ has been used in the industry of orthopedic medical devices; being part of the formulation of bone cements for bone repair, prosthesis manufacturing and as a component of controlled drug release systems [32]. Additionally, literature reports that this compound has been used for the improvement of the mechanical resistance of materials [33]. Therefore, it can be postulated that CaCO_3_ is responsible for the enhancement in the mechanical and thermal properties of the PECoVA copolymer. Furthermore, these XRD results suggest that the OMCD filler induced greater crystallinity in the PECoVA matrix compared to the DOL filler.

### 3.5. Mechanical Analysis by Tensile Test 

**Figure 6:**
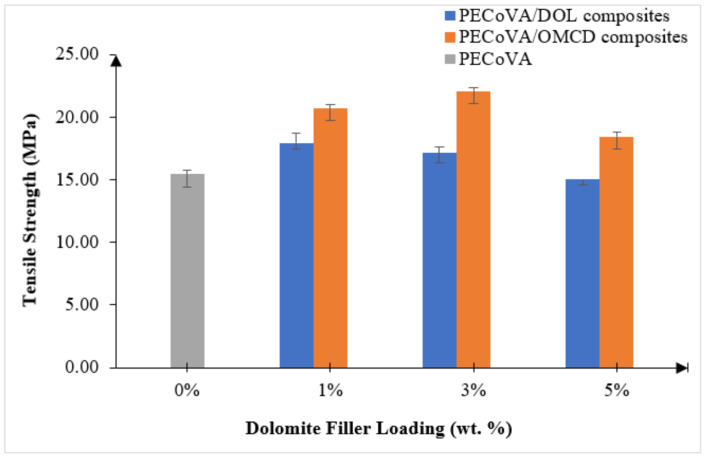
Effect of dolomite filler loading on the tensile strength of virgin PECoVA, PECoVA/DOL and PECoVA/OMCD composites.

**Figure 7:**
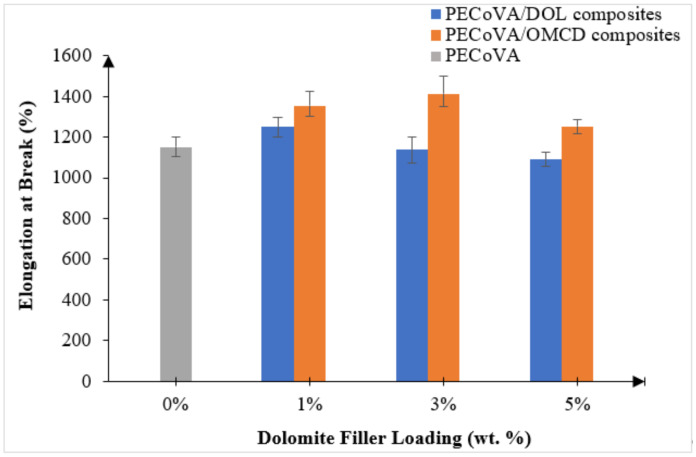
Effect of dolomite filler loading on the elongation at break of virgin PECoVA, PECoVA/DOL and PECoVA/OMCD composites.

A tensile test was conducted to determine the tensile strength, elongation at break and Young’s modulus of the materials. Table 5 summarizes the tensile properties of the virgin PECoVA, PECoVA/DOL and PECoVA/OMCD composites. Figure 6 and Figure 7 illustrate the effect of dolomite filler loading on the tensile strength and elongation at break of the virgin PECoVA, PECoVA/DOL and PECoVA/OMCD composites.

Based on the results, the addition of DOL and OMCD successfully improved the tensile strength and elongation at break of the PECoVA. In the presence of 1 wt% DOL, the PECoVA composites show an increase in tensile strength and elongation at break. However, as the percentage of DOL filler present in the composites increased from 1 to 5 wt%, the tensile strength and elongation at break of the PECoVA composites exhibit a decreasing trend. This pattern of changes indicates that the strength of PECoVA/DOL composites is compromised at high DOL filler loading. Agglomeration of dolomite leads to the formation of large particles that are not easily mixed with the PECoVA during the melt compounding process. This encouraged filler–filler interactions rather than filler–matrix interactions. 

The tensile behavior of the PECoVA/OMCD composites was expected to improve with the addition of the OMCD filler until it reached an optimum filler loading. Theoretically, mechanical performance of the polymer composites depends on the interfacial adhesion between matrix and filler [34]. The tensile strength and elongation at break of the PECoVA/OMCD composites were greatly improved by the addition of up to 3 wt% of OMCD, before dropping off at 5 wt% OMCD loading. The tensile strength and elongation at break of the virgin PECoVA were 15.4 MPa and 1151%, respectively. By the addition of as low as 1 wt% OMCD, the former and the latter values increased to 20.7 MPa and 1354%, respectively, which are equivalent to 34% and 18% increases as compared to the virgin PECoVA. The enhancement in tensile strength, stiffness and elongation at break of the composites is attributed to the reinforcement provided by the better interfacial adhesion and better dispersion of OMCD through the matrix. The chemical treatment and the size reduction process assist the dispersion of the OMCD and increase its surface area for greater PECoVA–filler interactions. The OMCD allows itself to occupy the free volume between the polymer chains and restrict the chain mobility, which eventually increase the energy required to permanently deform or break the composites [4,35]. The existence of stearic acid, which acted as organic surfactant in the PECoVA composites, also contributes to the enhancement in both tensile strength and elongation at break of the copolymer matrix. The coating of a monolayer of organic surfactant rendered the dolomite organophilic, meaning that it possessed similar polarity with the PECoVA, allowed for an improvement of interfacial adhesion between the PECoVA and filler. The OMCD with stronger PECoVA–filler interaction can hold the PECoVA chain strongly, which resulted in extra extension force.

The high elongation at break demonstrated by the PECoVA/OMCD3 composite might be because the smaller size of the OMCD filler increased its surface area contact with the matrix and thus more homogeneity in the PECoVA matrix. The mechanism behind this was the stimulation of chain relaxation of the PECOVA chain due to the inclusion of the OMCD in the PECOVA molecular chains. The attachment of the ester group of the surfactant to the surface of the dolomite caused disentanglement of some part of the copolymer chains, which generated more free volume that can provide more conformational freedom for molecular mobility. Subsequently, chain relaxation could be stimulated at the region of high stress concentration. As a result, the copolymer could achieve higher elongation before it breaks.

At higher OMCD content, which is at 5 wt%, the reduction in tensile strength and elongation at break is the result of the poor dispersion of the OMCD filler. In fact, due to high surface energy and extremely high aspect ratio at high content, they tend to create aggregate domains, leading to stress concentration at some points in the matrix during the tensile deformation. Consequently, the mechanical properties reduced. Similar findings have been reported by other researchers regarding the different polymer composites containing calcite- and carbonate-based fillers [36,37,38]. These studies also unanimously indicated that poor dispersion at high filler loading is responsible for deterioration of the mechanical properties.

**Figure 8:**
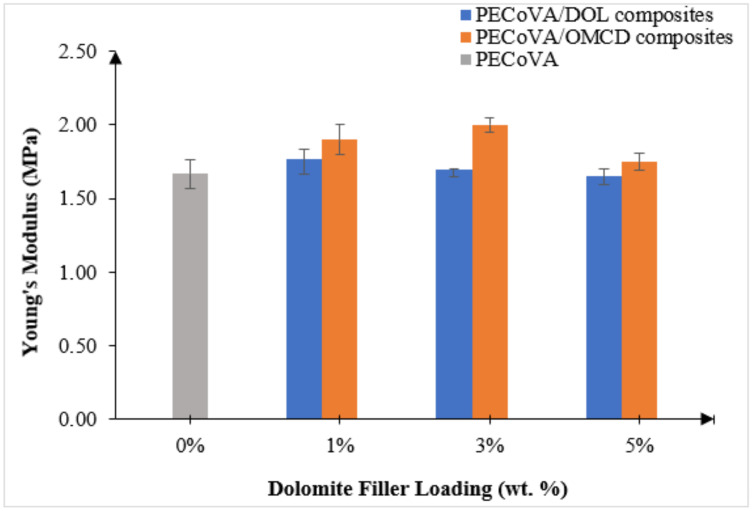
Effect of dolomite filler loading on the Young’s Modulus of virgin PECoVA, PECoVA/DOL and PECoVA/OMCD composites.

The changes in Young’s modulus were identified for both PECoVA/DOL and PECoVA/OMCD composites, as shown in Figure 8. It was found that the OMCD improved the Young’s modulus of the PECoVA, while the DOL resulted in the opposite effect. Young’s modulus of the PECoVA/DOL composite slightly increased to 1.8 MPa after the initial incorporation of 1 wt% of DOL into the PECoVA. Then, the Young’s modulus of the PECoVA/DOL composite decreased from 1.8 to 1.7 MPa after further incorporation of DOL (3 wt%) into the matrix. The Young’s modulus continuously reduced until the DOL content was at its highest loading, which is 5 wt%. The percentage of reduction for DOL loading was around 7%. On the other hand, the Young’s modulus of the PECoVA continuously increased and it was the highest when OMCD content was 3 wt%. The increment was because the mobility of the PECoVA chain was restricted by increasing the OMCD filler content. The stiffness of polymer matrices can be increased by the incorporation of rigid particulate filler as well as the interfacial contact that exists between the filler and matrix [39]. The improvement in Young’s modulus proved that the OMCD has the ability to stiffen the composites. Normally, a mineral filler produces a larger increase in Young’s modulus if it has a high aspect ratio (average equivalent diameter of the filler particle divided by its average thickness). Interestingly, even though the aspect ratio of OMCD is low (L/D = 3:1), it does help the PECoVA to improve its stiffness. This is also similar to Wiebking studies that reported precipitated CaCO_3_ with a low aspect ratio increasing the Young’s modulus of polyvinyl chloride compared to talc, which has a higher aspect ratio (L/D = 20:1) [40].

However, the Young’s modulus was reduced after the addition of 5 wt% OMCD. Although it is expected that the Young’s modulus will increase with increasing filler content, the agglomeration of filler can cause the presence of voids between the fillers and polymer matrix due to agglomeration of fillers at high filler content. It is believed to be the reason for the reduction in Young’s modulus with the addition of 5 wt% OMCD.

### 3.6. Mechanical Analysis by Tear Test

**Figure 9:**
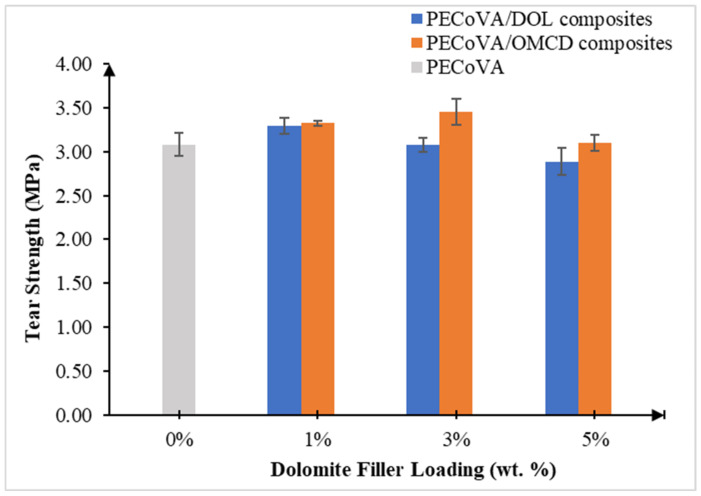
Effect of dolomite filler loading on the tear strength of virgin PECoVA, PECoVA/DOL and PECoVA/OMCD composites.

Tear test was performed to determine the tear strength of the materials. Table 6 indicates the tear strength of the virgin PECoVA, PECoVA/DOL and PECoVA/OMCD composites. Figure 9 illustrates the effect of dolomite filler loading on the tear strength of the PECoVA/OMCD and PECoVA/DOL composites.

Generally, the tear strength of the virgin PECoVA (3.083 MPa) is lower than that of the PECoVA/DOL and PECoVA/OMCD composite. This indicates that both the DOL and OMCD fillers successfully improved the tear strength of the PECoVA. However, the copolymer requires different optimum filler loadings when using DOL and OMCD as fillers for the production of the PECoVA composite with improved tear strength. For instance, the PECoVA/DOL composite achieved the highest tear strength when 1 wt% of filler was employed, while PECoVA/OMCD achieved the highest tear strength with the addition of 3 wt% of filler. The PECoVA/OMCD3 composites show the highest tear strength (3.456 MPa) among all the samples. It achieved 12% higher tear strength when compared with the virgin PECoVA. Obviously, when the same weight percentage of filler was used, the PECoVA/OMCD composites had higher tear strength than the PECoVA/DOL composites. This enhancement happened due to the surface treatment and the size reduction process, which help to homogenously distribute and disperse the OMCD particles into the PECoVA molecular chains and improve the matrix–filler interactions. As a result, increment in the stiffness and toughness of the matrix can be achieved when force is applied.

Based on Figure 9, the addition of 1 wt% DOL into the PECoVA resulted in an increase of the tear strength to 3.298 MPa, which is equivalent to a 6.97% increase as compared to the virgin PECoVA. As the weight percentage of DOL filler present in the composites further increased, the tear strength of the PECoVA/DOL composites continuously dropped until 2.886 MPa at its highest loading, which is 5 wt%. This might be due to the poor interfacial interaction between the polyethylene (PE) phase of the copolymer and the DOL filler, as both of them have different surface polarity and permeability. Normally, the molecules are only attracted by other molecules with similar polarity. DOL contains polar molecules (hydrophilic) which have positive and negative ends attracted to each other. Therefore, the DOL filler has high surface tension. On the other side, the non-polar PE molecules (hydrophobic) are not strongly attracted to each other as much as in the polar DOL molecules. Instead, these non-polar PE molecules are much less likely to have high surface tension. Therefore, the drop in the tear strength of the PECoVA/DOL composites explained that the non-polar DOL and polar PE molecules were impermeable/repelling each other. Although the DOL filler can strongly bond to the VA molecules of PECoVA due to the similar polarity, the VA content was only 21% (minor part) which was only able to contribute a small amount of reinforcing effect, as shown by the PECoVA/DOL1 composites. These observations also support the tensile test results. Similar findings have been reported by other researchers regarding the poor interaction between dissimilar polarity of matrix–filler in the composite systems [14,38]. Hence, it was assumed that the tear strength of the composites will continuously drop as the DOL content increased. The phenomena observed in the tear analysis are very similar to those observed in the tensile analysis, allowing us to double-check the material’s behavior and properties.

The homogenous distribution and dispersion of OMCD can increase the efficiency of filler to transfer the load within the PECoVA composite structure. From the results obtained, the tear strength data of the composite seem to follow the trend of the tensile strength data, in which the higher loading of OMCD filler causes a better reinforcing effect. The following statement explains this phenomenon: When a greater amount of OMCD particles penetrated through the polymer chains, they would develop more surface interactions with the PECoVA matrix; therefore, a better reinforcing effect can be seen. This is translated through the tensile and tear strength values. Osman, Edwards and Martin have reported a similar phenomenon in their composite system with mineral filler [41].

### 3.7. Thermomechanical Analysis by Dynamic Mechanical Analysis (DMA)

#### Thermomechanical Properties of the Virgin PECoVA, PECoVA/OMCD and PECoVA/DOL Composites

**Figure 10:**
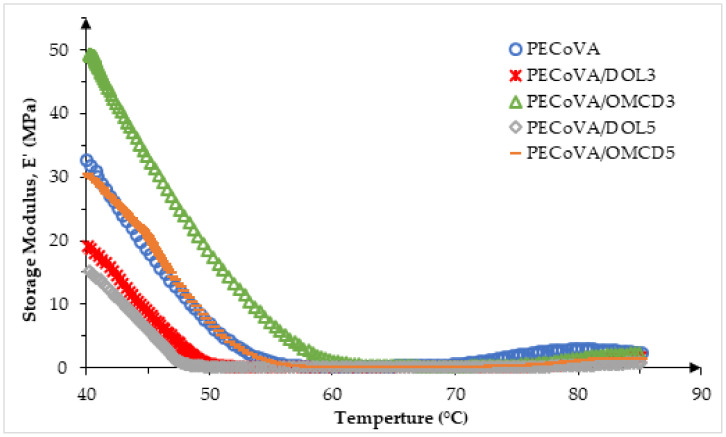
Effect of dolomite filler loading on the storage modulus, E’ of virgin PECoVA, PECoVA/DOL and PECoVA/OMCD composites.

Dynamic mechanical analysis (DMA) measures the response of virgin PECoVA, PECoVA/OMCD and PECoVA/DOL composites to an oscillatory deformation as a function of temperature. The results of DMA are usually analyzed by considering three viscoelastic properties such as storage modulus (E’), loss modulus (E”), and damping capacity (tan δ), which were evaluated as a function of temperature. Damping capacity (tan δ) is defined as the ratio of the loss modulus to storage modulus (E”/E’) [42]. It is used to determine the damping properties of the material which give the correlation between the elastic phase and viscous phase in a polymeric structure [43,44,45]. The measurement of the damping behavior is useful to determine the occurrence of molecular mobility transitions, such as the glass transition temperature (T_g_) or vicat softening temperature (T_Vicat_) of the PECoVA copolymer and its composites. DMA has been increasingly used to study the relationships between the polymer structure, dispersed phase as well as the calcite structure in the properties of polymer composites systems [42,46,47]. The maximum damping capacity (Tan δ_max_) and the T_Vicat_ are given in Table 7, while the E’ and tan δ curves analyzed by DMA are presented in Figure 10 and Figure 12, respectively.

The previous researches proved that the thermo-mechanical properties of the given materials are strongly affected by the interfacial adhesion between the polymer–filler and the dispersion of the filler in the polymer matrix [42,46,47]. When comparing the mechanical test (tensile and tear test) and DMA results of the virgin PECoVA, PECoVA/DOL and PECoVA/OMCD composites, one could agree that the treated OMCD filler gives more significant effect to the thermo-mechanical properties than the ambient mechanical properties of the host PECoVA. In fact, the OMCD influenced the thermo-mechanical properties of the PECoVA more than the DOL filler.

**Figure 11:**
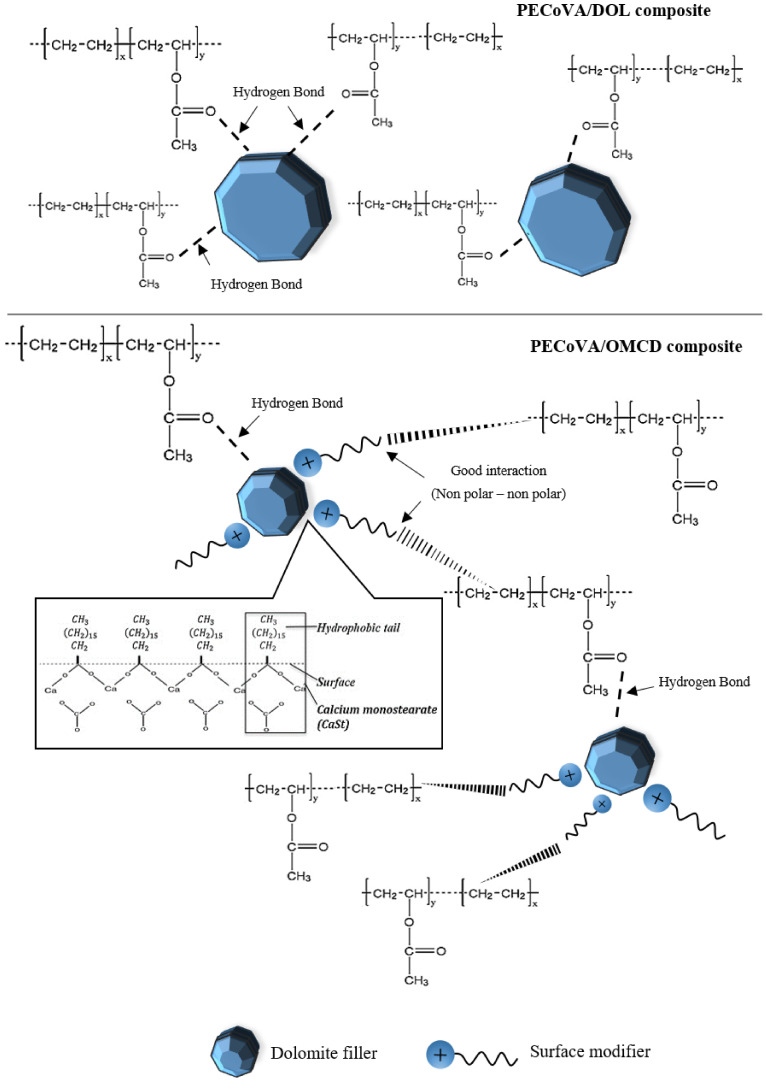
Proposed interactions between the PECoVA copolymer chains with DOL and OMCD filler in the PECoVA composite system.

Figure 10 illustrates the dynamic storage modulus (E’) as a function of temperature for the virgin PECoVA, PECoVA/DOL and PECoVA/OMCD composites. The value of storage or elastic modulus (E’) signifies the stiffness of the material corresponding to deformation’s elastic response [48,49]. It is widely known that the interaction between polymer–filler enhanced the storage modulus value of a polymer composite, at the high temperature rubbery zone, due to the larger deviations between the thermodynamic characteristics of the matrix and filler at high temperature range [50]. The improvements in stiffness observed in the PECoVA composites can be credited to the high stiffness behavior of the OMCD filler that will effectively constrain the movement of the PECoVA copolymer chains. It is clearly observed that the PECoVA/DOL5 composite has the lowest storage modulus, while the virgin PECoVA exhibits the intermediate storage modulus over the analyzed temperature range. Apparently, the PECoVA composites containing the OMCD (PECoVA/OMCD3 and PECoVA/OMCD5) exhibit greater storage modulus as compared to the PECoVA composites containing the pristine DOL (PECoVA/DOL3 and PECoVA/DOL5). These show that the PECoVA/OMCD3 composites are stiffer than the virgin PECoVA, PECoVA/DOL3 and PECoVA/DOL5 when conditioned at a temperature range between room temperature to 85 °C. This could be associated with good interfacial adhesion between the PECoVA and the OMCD, and good dispersion of the filler throughout the PECoVA matrix, as the OMCD had been surface modified and tip sonicated prior to mixing with the PECoVA copolymer. This caused the improvement in PECoVA/OMCD interactions that led to stiffening of the host PECoVA copolymer chains. Specifically, the physical molecular interaction via hydrogen bonds between polar groups of the PECoVA with carbonyl groups of the stearic acid, and non-polar interactions between the PE phase of the PECoVA with the organic surfactant (stearic acid), resulted in stiffening of the PECoVA matrix. Figure 11 illustrates the proposed PECoVA–filler interactions in the PECoVA composite systems containing the DOL versus the OMCD. Initially, the DOL is comprised of crystalline particles that have affinity toward each other; they stacked and formed aggregates. Thus, the PECoVA composite with the pristine DOL contains lower degrees of filler distribution and dispersion. The presence of large aggregates causes less interaction between the PECoVA copolymer chains and the DOL particles (Figure 3b). In contrary, the OMCD contains smaller size particles with organophilic surface. This facilitates the dispersion of OMCD particles to occupy the free volume between the PECoVA chains (please refer to the XRD result where diffraction signals of OMCD can be detected in the PECoVA/OMCD composite). A more homogeneous composite was obtained when the OMCD was employed as filler (FESEM result—Figure 3c). More interactions between the matrix and filler are obtained due to: (i) Hydrogen bond between the vinyl acetate phase of the PECoVA and hydroxyl group of the dolomite surface (please refer to the FTIR and DMA results); (ii) non-polar–non-polar interactions between the hydrocarbon tail of the surface modifier’s chains (stearic acid) and polyethylene phase of the PECoVA (please refer to the XRD result (crystallinity index). This contributes to the excellent reinforcing capability of the OMCD towards the PECoVA matrix (please refer to tensile and tear results). Without the surface modification and tip sonication process, the resultant PECoVA composite would not achieve an appreciable enhancement in the storage modulus over the virgin PECoVA. Poor dispersion of DOL would not encourage significant polymer–filler interactions. The storage modulus data of the PECoVA/DOL5 sample proved this statement.

**Figure 12:**
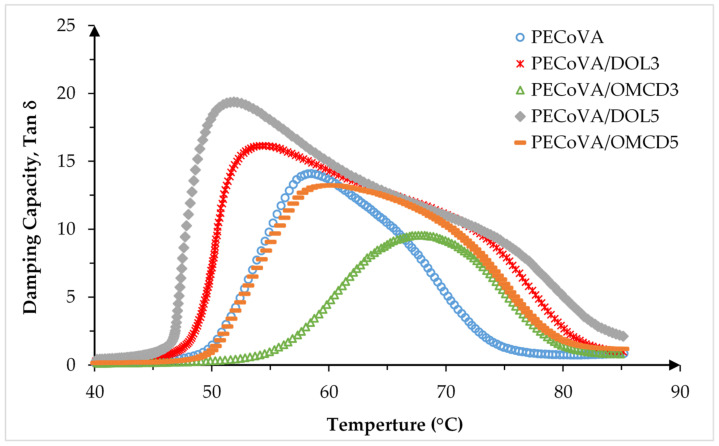
Effect of dolomite filler loading on the damping capacity (Tan δ) of virgin PECoVA, PECoVA/DOL and PECoVA/OMCD composites.

The graph in Figure 12 reveals the damping capacity (Tan δ) of the virgin PECoVA, PECoVA/DOL and PECoVA/OMCD composites in the range of room temperature until 90 °C. It is noticed that when temperature increased, the Tan δ increased to the maximum value (Tan δ_Max_) in the transition region and decreased in the rubbery region. It is related to the movements of molecules and small groups within the copolymer structure of PECoVA [49,50]. The Tan δ_Max_ and T_Vicat_ of the virgin PECoVA were 14.0920 and 58.52 °C, respectively. This peak transition ranging from 45 to 58 °C corresponds to Vicat softening temperature for PECoVA [51]. Furthermore, the virgin PECoVA possesses a damping peak between the PECoVA/DOL and PECoVA/OMCD composites, indicating the intermediate degree of molecular mobility as compared to both types of the composites. All of the OMCD-filled PECoVA composites (PECoVA/OMCD3 and PECoVA/OMCD5) show significantly higher T_Vicat_ with comparatively lower Tan δ, while the increase of DOL filler loading (from 1 to 5 wt%) in the PECoVA shifted up the Tan δ_Max_ and simultaneously reduced the T_Vicat_ of PECoVA/DOL composites. These are due to the integration of OMCD filler particles that decreases the viscoelastic damping factor of the PECoVA matrix. The decrease of Tan δ_Max_ proposes that the process for energy dissipation becomes slower by the addition of OMCD filler. This was due to the presence of the OMCD filler that restricted the molecular mobility of the PECoVA chains. PECoVA/DOL composites show comparatively lower T_Vicat_, which can confirm the weaker interaction of the DOL surface with PECoVA chains. The DMA results show that the addition of 3 wt% of OMCD successfully lowered the Tan δ_Max_ and enhanced the T_Vicat_ of PECoVA composites to 9.5569 and 67.91 °C, respectively. However, further increase in the OMCD loading to 5 wt% will increase the Tan δ_Max_ of the composites, suggesting the weakening of the matrix–filler interaction due to poor dispersion of filler in the PECoVA matrix. Thus, it is believed that the incorporation of 3 wt% filler loading into the virgin PECoVA is already enough for the composite to have the optimum interfacial adhesion, because the strong bonded interface of the composite will reflect a low magnitude of Tan δ_Max_ and higher T_Vicat_ [45]. Other than the DMA results, better dispersion of OMCD particles in the composites obtained after the combination of sonication and surface treatment can be also proven by DSC analysis.

The PECoVA/OMCD3 composite indicates a lower Tan δ_Max_ that appears at a higher temperature than that of PECoVA/OMCD5, virgin PECoVA PECoVA/DOL3 and PECoVA/DOL5. The main reason was due to the existence of more restricted PECoVA chain mobility in the PECoVA/OMCD3 composite, caused by greater PECoVA–OMCD interactions. Previous research also implied that this Tan δ_Max_ that shifted to a somewhat broader peak with a higher temperature was due to more molecular mobility restriction forced by dispersed OMCD in the PECoVA molecular chains [52]. The results also indicated that the DOL loading brought significant influence on the damping behavior of the PECoVA composites. When the filler loading increased from 3 to 5 wt% (DOL), an increase in Tan δ_Max_ intensity was perceived. This was due to weaker matrix–filler interactions as a result of reduced quality of DOL dispersion in the PECoVA matrix.

An anomalous behavior was observed when the PECoVA was added with (3 and 5 wt%) DOL filler. The presence of a more intense and broader peak can be observed through the DMA curve. This may be because the hydrophilic DOL filler, that induced the phase separation in the PECoVA system at the weaker interphase site between the VA and PE molecules, resulted in a more isolated PE structure.

From the obtained results, it is clear that the PECoVA composites containing the OMCD filler had better tensile and tear properties as compared to the PECoVA composites containing the pristine DOL. As proved through the SEM and XRD results, this was due to the homogeneous distribution, improved filler dispersion and good interfacial adhesion of OMCD inside the PECoVA matrix as a result of tip sonication and surface treatment process. Without these processes, the dolomite could not serve as an efficient reinforcing filler. As proved through the mechanical data, the tensile strength and toughness of the PECoVA/DOL composite showed a decreasing trend when the DOL content increased from 1 to 5 wt%. In contrast, the tensile strength of the PECoVA/OMCD composite increased more significantly when 1 wt% filler was employed and further increased when the filler content increased to 3 wt%.

In this study, the DMA analysis was conducted in the range of room temperature to 85 °C. Based on the supplier datasheet, the primary relaxation temperature (T_α_) which corresponds to glass transition temperature (T_g_) of this PECoVA copolymer is around 70 °C. However, due to the unavailability of liquid nitrogen during this DMA measurement, the experiment could not be started from minus degree Celsius, thus the presence of T_α_ was not observed.

### 3.8. Thermal Behavior by Differential Scanning Calorimetry (DSC)

**Figure 13:**
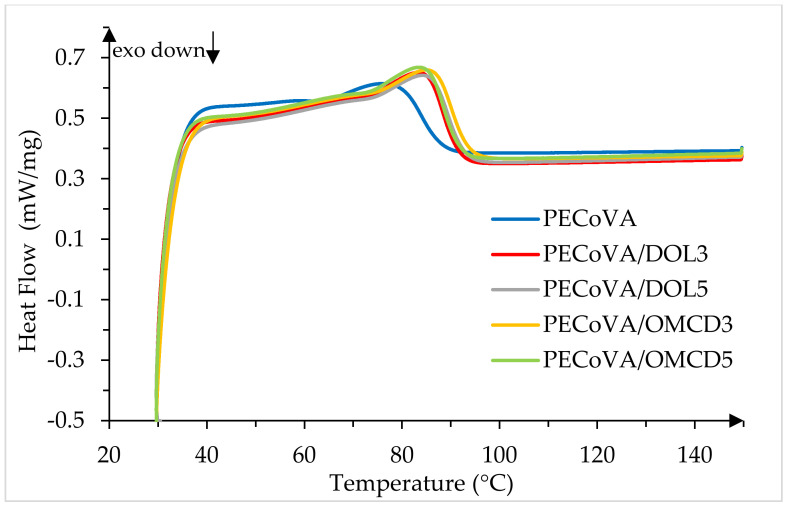
Effect of dolomite filler loading on the melting temperature, T_m_, of virgin PECoVA, PECoVA/DOL and PECoVA/OMCD composites.

The thermal behavior of the virgin PECoVA, PECoVA/OMCD and PECoVA/DOL composites were studied by differential scanning calorimetry (DSC), which was applied to determine the thermal properties such as phase transition (glass transition or melting transition) and changes in heat capacity of polymers during the heating or cooling procedures. The peak of melting temperature (T_m_), enthalpy of melting (Δ*H_f_)* and crystallinity (*X_c_*) are recorded in Table 8, while the melting curves analyzed by DSC are presented in Figure 13. Generally, the PECoVA copolymers only exhibit two transition states, which are glass transition temperature (T_g_) and T_m_; therefore, heating the polymer beyond T_g_ will result in a positive jump in the heat flow (melting transition) with melting point. PECoVA is theoretically random copolymers, and thus the T_m_ should be single and will usually fall between those of the corresponding homopolymers [53] (melting point for polyethylene: 110–120 °C; softening temperature for poly(vinyl acetate): 35–50 °C).

Figure 13 illustrates the DSC plot of virgin PECoVA, PECoVA/OMCD and PECoVA/DOL composites, showing the heating curves (heat flow versus temperature) obtained in the second heating run with a constant heating rate of 10 °C·min^−1^ that were used for determination of T_m_. T_m_ is a first-order transition, which is normally observed as endothermic peak maximum in DSC heating curves. The DSC graph clearly shows one endothermic transition in each type of sample. Initially, the T_m_ of the virgin PECoVA was 78.6 °C. The addition of either DOL or OMCD filler into the PECoVA copolymer clearly enhanced the thermal properties of the PECoVA copolymer. When the filler loading increased from 3 to 5 wt%, the T_m_ of the PECoVA/DOL composites slightly decreased from 83.6 to 83.3 °C, while T_m_ of PECoVA/OMCD slightly increased from 84.3 to 84.8 °C. Obviously, the PECoVA/DOL composites show endothermic transitions up to ~83 °C; however, beyond ~83 °C, transitions become exothermic in nature. However, the PECoVA/OMCD composites show an exothermic transition at ~84 °C, indicate that, at a relatively higher temperature, they divert to being endothermic in nature. The shift in the heat flow means getting more heat and thus increasing the heat capacity of the polymer. Interestingly the intensity of the endothermic transition is found to be more intense for 3 wt% of OMCD filler among all the PECoVA composites. This means the PECoVA composites with 3 wt% of OMCD have the highest heat capacity among all samples. The higher increase of T_m_ and heat flow for PECoVA/OMCD3 composites, in comparison to PECoVA/DOL3 composites, predicts that the restriction of the mobility of PECoVA polymer chains are more precise as a result of the homogeneous distribution and dispersion of OMCD filler in the PECoVA matrix. Thus, the inclusion of OMCD filler in the PECoVA matrix restricted the polymer chains movement, either through physical or chemical interactions, which increases the T_m_ and heat capacity of the PECoVA copolymer. Consequently, thermal analysis, including DMA and DSC results, revealed that 3 wt% is the optimum OMCD filler loading to fabricate thermally-stable PECoVA composites, as it offers a virtuous resistance or stability towards heat in the PECoVA copolymer.

The endothermic transition between 75–95 °C caused an increase in the kinetic energy of molecules and the polymer chains began to fall apart between each other, that is, they melt. The polymer chains come out of their ordered arrangements and begin to move around freely, thus induce the polymer melt to flow [54]. In order to melt the PECoVA crystals, they must absorb energy (heat). When polymer reaches the T_m_, the polymer’s temperature will not rise until all the crystals have melted. Accordingly, the little heater under the sample pan has to supply the PECoVA composites with a lot of heat in order to melt the crystals and to keep temperature rising at the same rate as that of the reference pan. The heat flow during melting shows as a peak on the DSC plot in Figure 13.

Similarly, the crystallinity confirms the difference of the crystal perfection of these composites. The degree of crystallinity for PECoVA/DOL3 and PECoVA/DOL5 is only 12.60% and 12.56%, respectively, which is no more than 12.69% of PECoVA/OMCD3 or no more than 12.82% of PECoVA/OMCD5. Table 8 shows that PECoVA/OMCD3 possesses the highest value of degree of crystallinity, suggesting that the OMCD with reduced particle size and improved surface compatibility can perfectly squeeze into the microscopic pores that exist in the PECoVA matrix, and tend to crystalline the composites more perfectly than the untreated DOL filler. It is worth noting that the onset melting temperatures of these copolymers are difficult to discern because of a small change in heat capacity and broad melting range, indicating all of these PECoVA composites have a wide size distribution of either the PE or filler crystallites [55]. With the similar trend from DMA and XRD results, the PECoVA copolymer with OMCD filler gives higher crystallinity compared to the DOL filler due to fine particles size, homogenous dispersion and better interfacial interaction between polymer matrix and filler. 

The DSC analysis was carried out in this study at temperatures starting from ~30 to 150 °C. The T_g_ of this PECoVA copolymer is around −70 °C according to the material datasheet. However, previous studies also stated that it was impossible to obtain T_g_ of PECoVA copolymers by DSC analysis, either because of a small change in heat capacity or because the crystalline phase due to the PE contained in the copolymers might hide this phenomenon [51].

## 4. Conclusions

In this work, the combination of planetary ball mill and tip sonication were used to successfully reduce the particle size and de-agglomerate the dolomite filler. Then, the size-reduced dolomite was subjected to surface modification using stearic acid to obtain an “organophilic” filler. The changes in structure and morphology of the modified dolomite were witnessed and confirmed through FTIR and SEM analyses. We demonstrated that the size-reduced microcrystalline dolomite coated with organophilic surface (OMCD) possessed a better morphology and crystallinity than the pristine/untreated dolomite filler (DOL). This modified dolomite successfully improved the mechanical and thermal properties of the PECoVA copolymer when added in at 3 and 5 wt% as the filler phase of the composite. The results showed that the planetary ball mill, tip sonication and surface treatment successfully improved the dispersion of the dolomite filler in the copolymer matrix, thus enhanced the morphology, mechanical and thermal properties of the PECoVA matrix. The highest mechanical and thermal properties were achieved when the PECoVA was incorporated with 3 wt% of OMCD. This composite system exhibits more homogenous mixing and good interfacial interaction between the filler and the copolymer matrix.

## Figures and Tables

**Table 1:** Formulation and acronyms of PECoVA/dolomite composites.

PECoVA (wt%)	OMCD (wt%)	DOL (wt%)	Acronym
100	-	-	PECoVA
99	1	-	PECoVA/OMCD1
99	-	1	PECoVA/DOL1
97	3	-	PECoVA/OMCD3
97	-	3	PECoVA/DOL3
95	5	-	PECoVA/OMCD5
95	-	5	PECoVA/DOL5

DOL = Pristine Dolomite; OMCD = Organophilic Microcrystalline Dolomite.

**Table 2:** Surface area and particle size of (DOL), (DOL+10H) and (DOL+10H+2H).

Samples	Surface Area (m^2^)	Particle Size (m)
DOL	2281 ± 239	58 ± 6
DOL+10H	729 ± 135	29 ± 2
DOL+10H+2H	180 ± 17	15 ± 4

**Table 3:** Crystallinity of the DOL and OMCD based on XRD analysis.

Samples	Percent (%)	
	Crystallinity	Amorphous
DOL	69.19	30.81
OMCD	71.86	28.14

**Table 4:** Crystallinity of the virgin PECoVA, PECoVA/DOL3 and PECoVA/OMCD3 composites based on XRD analysis.

Samples	Percent (%)	
	Crystallinity	Amorphous
PECoVA	23.29	76.71
PECoVA/DOL3	23.69	76.31
PECoVA/OMCD3	34.72	65.28

**Table 5:** Tensile properties of virgin PECoVA, PECoVA/DOL and PECoVA/OMCD composites.

	Filler Loading (wt%)	Sample		
		PECoVA	PECoVA/DOL	PECoVA/OMCD
**Tensile Strength (MPa)**	0	15.4 ± 2	-	-
	1	-	18.0 ± 1	20.7 ± 1
	3	-	17.1 ± 1	22.1 ± 1
	5	-	15.0 ± 1	18.5 ± 1
**Elongation at break (%)**	0	1151 ± 47	-	-
	1	-	1249 ± 49	1354 ± 68
	3	-	1150 ± 65	1413 ± 87
	5	-	1090 ± 35	1252 ± 34
**Young’s Modulus (MPa)**	0	1.7 ± 0.1	-	-
	1	-	1.8 ± 0.1	1.9 ± 0.1
	3	-	1.7 ± 0.1	2.0 ± 0.1
	5	-	1.6 ± 0.1	1.8 ± 0.1

**Table 6:** Tear strength of virgin PECoVA, PECoVA/DOL and PECoVA/OMCD composites.

Type of Sample	Tear Strength (MPa)
PECoVA	3.083 ± 0.08
PECoVA/DOL1	3.298 ± 0.09
PECoVA/DOL3	3.081 ± 0.08
PECoVA/DOL5	2.886 ± 0.16
PECoVA/OMCD1	3.325 ± 0.03
PECoVA/OMCD3	3.456 ± 0.15
PECoVA/OMCD5	3.104 ± 0.09

**Table 7:** Damping capacity of virgin PECoVA, PECoVA/DOL and PECoVA/OMCD composites.

Type of Sample	Maximum Damping Capacity, Tan δ_Max_	Vicat Softening Temperature, T_Vicat_ (°C)
PECoVA	14.1	58.5
PECoVA/DOL3	16.2	54.4
PECoVA/DOL5	19.4	51.9
PECoVA/OMCD3	9.6	67.9
PECoVA/OMCD5	13.2	60.1

**Table 8:** Melting temperature of virgin PECoVA, PECoVA/DOL and PECoVA/OMCD composites.

Type of Sample	T_m_ (°C)	Δ*H_f_* (J/g)	*X_C_* (%)
PECoVA	78.6	31.92	11.52
PECoVA/DOL3	83.6	34.91	12.60
PECoVA/DOL5	83.3	34.81	12.56
PECoVA/OMCD3	84.8	35.51	12.82
PECoVA/OMCD5	84.3	35.15	12.69

T_m_: Peak melting temperature; Δ*H_f_*: Enthalpy of melting/fusion; *X_C_*: Crystallinity; Δ*H_f_^0^*: Polyethylene is 277.1 J/g.

## Data Availability

The data presented in this study are available on request from the corresponding author.
